# Spliced or Unspliced, That Is the Question: The Biological Roles of XBP1 Isoforms in Pathophysiology

**DOI:** 10.3390/ijms23052746

**Published:** 2022-03-02

**Authors:** Xinxin Luo, Leader Alfason, Mankun Wei, Shourong Wu, Vivi Kasim

**Affiliations:** 1 Key Laboratory of Biorheological Science and Technology, Ministry of Education, College of Bioengineering, Chongqing University, Chongqing 400044, China; xinxinluo59@163.com (X.L.); leadalfa214@126.com (L.A.); weimankun@126.com (M.W.); 2 The 111 Project Laboratory of Biomechanics and Tissue Repair, College of Bioengineering, Chongqing University, Chongqing 400044, China; 3 State and Local Joint Engineering Laboratory for Vascular Implants, Chongqing 400044, China

**Keywords:** unspliced XBP1 (XBP1-u), spliced XBP1 (XBP1-s), transcriptional activator, post-translational modification, physiological and pathological pathways

## Abstract

X-box binding protein 1 (XBP1) is a member of the CREB/ATF basic region leucine zipper family transcribed as the unspliced isoform (XBP1-u), which, upon exposure to endoplasmic reticulum stress, is spliced into its spliced isoform (XBP1-s). XBP1-s interacts with the cAMP response element of major histocompatibility complex class II gene and plays critical role in unfolded protein response (UPR) by regulating the transcriptional activity of genes involved in UPR. XBP1-s is also involved in other physiological pathways, including lipid metabolism, insulin metabolism, and differentiation of immune cells. Its aberrant expression is closely related to inflammation, neurodegenerative disease, viral infection, and is crucial for promoting tumor progression and drug resistance. Meanwhile, recent studies reported that the function of XBP1-u has been underestimated, as it is not merely a precursor of XBP1-s. Instead, XBP-1u is a critical factor involved in various biological pathways including autophagy and tumorigenesis through post-translational regulation. Herein, we summarize recent research on the biological functions of both XBP1-u and XBP1-s, as well as their relation to diseases.

## 1. Introduction

X-box binding protein 1 (XBP1), which belongs to the basic leucine zipper (bZIP) protein family, was first isolated in human B lymphoblastoid cell lines during a screening for key genes that regulate major histocompatibility complex (MHC) class II molecules using a human B cell cDNA expression library with an X-box target sequence [1]. The *XBP1* gene, which is highly conserved in mammals [2], is located at chromosome 22q12 in humans. It is translated into two isoforms, namely, unspliced XBP1 (XBP1-u) and spliced XBP1 (XBP1-s).

Since its discovery, a large number of studies regarding XBP1 have been performed. However, most of them involved spliced XBP1, or XBP1-s, but not XBP1-u. XBP1-s is produced upon exposure to endoplasmic reticulum (ER) stress resulting from changes in extrinsic and intrinsic factors, including microenvironment, overproduction of reactive oxygen species (ROS), excessive proliferation, and viral infection. This in turn induces the accumulation of unfolded or misfolded protein, and finally triggers the unfolded protein response (UPR). XBP1-s acts as a transcriptional factor that recognizes cis-acting sequences in the promoter regions of its target genes and regulates the transcription of a series of target genes involved in UPR [3,4,5]. Recent studies also revealed that XBP1-s is also crucial for the regulation of insulin and lipid metabolism by regulating cellular function and activating the transcriptional activity of fatty acid synthase (FAS) [6], respectively, as well as for the development of the immune system by regulating the terminal differentiation of plasma cells (PCs) and eosinophils. Furthermore, XBP1-s can aggravate inflammation by binding directly to the promoters of inflammatory factors such as *interleukin-8* (*IL-8*), *chemokine ligand 5* (*CCL5*), and *interferon β* (*IFN-β*), and can exacerbate viral infection by promoting viral DNA replication [7,8]. XBP1-s is also closely related to the induction of neurodegenerative disease through its regulation of motor and memory function. Moreover, high expression of XBP1-s positively correlates with tumor progression, poor prognosis, and drug resistance.

Unlike those on XBP1-s, studies regarding the pathophysiological functions of XBP1-u are still very limited. Since its discovery, XBP1-u has been assumed to be merely a precursor of XBP1-s, without its own, unique biological and pathological functions. This underestimation of XBP1-u function lasted for quite a long time, until Zhao et al. revealed that XBP1-u could suppress autophagy by enhancing forkhead box O1 (FoxO1) ubiquitination/proteasomal degradation [9]. Since then, emerging evidence has revealed that XBP1-u is not merely a precursor of XBP1-s, but is a critical factor in various biological pathways, such as protection of endothelial cells from oxidative stress, regulation of the cell cycle, and vascular calcification, and functions in an XBP1-s independent manner [10,11,12,13]. These studies pointed out the special, unique functions of XBP1-u through mechanisms distinct from those of XBP1-s.

In this review, we will outline the structural and functional differences between XBP1-u and XBP1-s, as well as their physiological and pathological functions, especially in the regulation of metabolism, differentiation, inflammation, neurodegenerative diseases, and tumor progression.

## 2. The Two Isoforms of XBP1

### 2.1. Structural Differences between XBP1-u and XBP1-s

While XBP1-s is the most studied XBP1 isoform, it is not a direct product of the *XBP1* gene. The *XBP1* gene, which contains 6010 base pairs, is transcribed as a precursor mRNA containing 7 exons and 5 introns. The introns are then spliced, producing XBP1-u mRNA with 1820 nucleotides containing 5 exons. The start codon of XBP1-u is located at +49 to +51 in exon 1, while the stop codon is located at +832 to +834 in exon 5, thus producing XBP1-u protein with 261 amino acids (28.71 kDa). The N terminus of XBP1-u contains a DNA binding domain; however, its C terminus contains a nuclear export signal (NES) and a degradation domain that could trigger its proteasomal degradation [14]. Indeed, XBP1-u is the major isoform of XBP1 under non-ER stress conditions [15].

**Figure 1:**
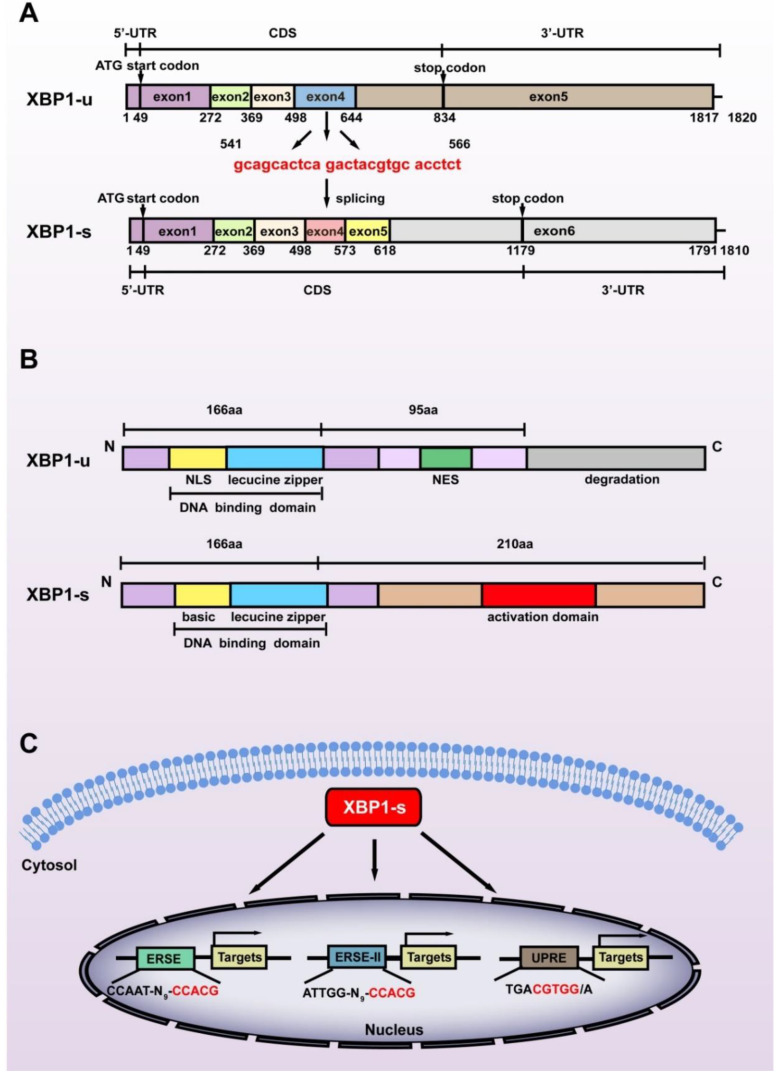
Structural differences between the two splicing isoforms of XBP1. (**A**) The difference between the nucleic acid sequences of XBP1-u and XBP1-s. The spliced sequences are shown in red. (**B**) The difference between the amino acid sequences of XBP1-u and XBP1-s. The C-termini of these two isoforms consist of totally different amino acid sequences due to the frameshift caused by splicing at +541 to +566 of XBP1-u mRNA. NLS: nuclear localization sequence; NES: nuclear export sequence. (**C**) The three main cis-acting elements recognized by XBP1-s are shown. The core binding sequences are shown in red. ERSE: endoplasmic reticulum stress response element; ERSE-II: endoplasmic reticulum stress response element II; UPRE: unfolded protein response element.

Exposure to stressors including hypoxic condition, nutrient deficiency, excessive ROS, increased metabolic requirements, and decreased adenosine triphosphate (ATP) production triggers the splicing of XBP1-u. Under non-stress conditions, chaperone binding immunoglobulin protein (BiP) binds with inositol-requiring protein 1α (IRE1α), PERK-like ER kinase (PERK), and activating transcription factor 6 (ATF6), which act as sensor membrane proteins and assist transmembrane transport as well as correct folding of transmembrane receptors [16]. Upon exposure to stress, BiP is titrated away from the sensors owing to its higher affinity to bind with misfolded proteins [17]. This is followed by IRE1α oligomerization in the ER membrane, which enables its autophosphorylation, which in turn leads to the activation of its RNase kinase domain. This activation in turn promotes the splicing of XBP1-u, excluding 26 nucleotides located at +541 to +566 of the XBP1-u mRNA, and causing a frameshift in the XBP1-s coding sequence (CDS) (Figure 1A). Hence, while possessing exactly the same amino acid sequences with XBP1-u before the splicing site, i.e., from the first to the 166th amino acid in their C termini, the C terminus of XBP1-s is totally different from that of XBP1-u. The frameshift also alters the position of the stop codon to +1177 to +1179 in XBP1-s CDS, resulting in a larger protein translated from XBP1-s mRNA (376 amino acids, 41.36 kDa) compared to that translated from XBP1-u mRNA (Figure 1B). Furthermore, it also leads to the formation of a transcriptional activator domain in the C terminus of XBP1-s, enabling it to regulate target gene transcription. As will be discussed below, the structural differences of the C termini of XBP1-u and XBP1-s lead to their distinct biological functions as well as distinct regulatory mechanisms on target genes.

### 2.2. XBP1-s Mediates Transcriptional Regulation

Due to the presence of the transcriptional activator domain in its C terminus, XBP1-s can function as a transcriptional regulator targeting a series of genes [18]. After being spliced in the cytosol, XBP1-s translocates into the nucleus and induces the transcription of its target genes by recognizing and binding mainly to one of the following cis-acting promoter motifs: unfolded protein response element (UPRE): TGACGTGG/A; endoplasmic reticulum stress response element (ERSE): CCAAT-N9-CCACG; and endoplasmic reticulum stress response element II (ERSE-II): ATTGG-N-CCACG, with CGTCC in the UPRE and CCACG in the ERSE as well as ERSE-II as its core binding motifs (Figure 1C) [3,4,5]. Unlike another crucial component of ER stress, ATF6, which binds to ERSE or ERSE-II in a nuclear factor Y (NF-Y)-dependent manner, XBP1-s binds to and regulates these elements independently [19]. Furthermore, while not well documented [20], Clauss et al. reported that XBP1-s preferentially binds to CRE-like element GATGACGTG(T/G)NNN(A/T)T, whose core sequence, ACGT, is highly conserved [21].

Binding of XBP1-s to the above-mentioned cis-acting sequences leads to the alteration of the expression of various genes (Table 1). XBP1-s binds to UPRE and induces the transcription of *a disintegrin and metalloproteinase 10 (ADAM10)*, *Krüppel-like factor 9* (*KLF9*), and *basic helix–loop–helix family member a15* (*BHLHA15*, also known as *MIST1*) [22,23,24]; while its binding to ERSE induces the transcription of ER chaperones such as glucose regulated protein 94 (GRP94), BiP, and calreticulin, as well as *peroxisome proliferator-activated receptor γ2* (*PPARγ2*) and *sterol regulatory element binding transcription factor 1* (*SREBP-1*) [4,25,26,27]. Meanwhile, ERSE-II binding motif could be found in *homocysteine-inducible ER protein with ubiquitin-like domain 1* (*Herpud1*) and *arginine rich, mutated in early stage of tumors* (*ARMET*) [3,28]. In addition to the above-mentioned three major binding motifs, XBP1-s regulates the transcription of the *protein disulfide isomerase (PDI) family*, *including protein disulfide isomerase P5 (P5), protein disulfide isomerase (PDI), protein disulfide isomerase related protein (PDIR)*, *endoplasmic reticulum protein 44 (ERp44)*, and *endoplasmic reticulum protein 46* (*ERp46*), as well as adipogenesis related gene *CCAAT/enhancer-binding protein α* (*C/EBPα*), by recognizing ACGT as the core sequence [29,30]. Moreover, XBP1-s binds to unfolded protein response element II (UPRE-II; ATTGG-CCGGT) and induces the transcription of *brain-derived neurotropic factor* (*BDNF*) [31]. XBP1-s can also bind to promoters of *protein kinase cAMP-dependent type II regulatory subunit β* (*PRKAR2B*; binding motif: ACGTCA) and *activating transcription factor 4* (*ATF4*; binding motif: AGGGAGACGGCAGC) [32,33].

### 2.3. XBP1-u Mediates Post-Translational Modification

XBP1-u protein is formed as a direct translational product of mature, unspliced XBP1 mRNA. While it possesses a nuclear localization sequence (NLS) and a DNA binding domain in its N terminus, which is conserved between the two XBP1 splicing isoforms, it also has a strong NES in its C terminus after the splicing site, making it localized at the cytoplasm and unable to enter the nucleus by itself. Furthermore, unlike XBP1-s, XBP1-u does not possess a transcriptional activator domain in its C terminus. These differences make XBP1-u unable to function as a transcriptional regulator. While Yoshida et al. reported that XBP1-u could interact with XBP1-s and alter its localization in the cytoplasm, leading to the proteasomal degradation of this complex and a complete closure of UPR-mediated transcription [14], there was no report regarding its unique, ER stress-independent functions. Thus, XBP1-u has been assumed to be merely a precursor of XBP1-s until recent studies reported its unique, specific functions independent of ER stress.

The first study regarding the specific function of XBP1-u revealed its regulation of autophagy. FoxO1 is a key factor in inducing autophagy under serum starvation or oxidative stress. Zhao et al. found that under glutamine starvation, extracellular regulatory protein kinase 1/2 (ERK1/2) could phosphorylate the Ser61 and Ser176 of XBP1-u, leading to the enhanced interaction of XBP1-u and FoxO1. XBP1-u then recruits FoxO1 to the 20S proteasome and degrades FoxO1 to inhibit autophagy, thereby suppressing tumor cell death [9].

XBP1-u is also involved in cellular redox homeostasis. Martin et al. revealed that disturbed flow (D-Flow) induces a complex formation among XBP1-u, mammalian target of rapamycin complex 2 (mTORC2), Akt serine/threonine kinase 1 (Akt1), and histone deacetylase 3 (HDAC3), thereby stabilizing XBP1-u and HDAC3. Overexpression of XBP1-u and HDAC3 activated mTOR-mediated Akt1 phosphorylation, leading to the stabilization of NF-E2-related factor 2 (Nrf2). Nrf2 then translocates into the nucleus, binds to the *heme oxygenase 1* (*HO-1*) promoter, and promotes its transcription. *HO-1* in turn protects cells from oxidative stress by catalyzing heme degradation, which protects cells from oxidative stress [10].

In 2017, our group reported another XBP1-u-induced post-translational modification. Through a high-throughput screening for factors regulating the p53/p21 axis, we identified XBP1-u as a novel regulator of p53. We showed that without exposure to ER stressors such as hypoxia and nutrient deficiency, XBP1-u is the major form of XBP1 in tumor cells and is crucial for regulating p53 protein stability at the post-translational stage. XBP1-u binds with mouse double-minute 2 homolog (MDM2), an E3 ligase that induces the ubiquitination of tumor suppressor p53 protein through its C terminus, a region that derives it from XBP1-s. This binding prevents the formation of MDM2 homodimer, thereby preventing MDM2 self-ubiquitination that would lead to MDM2 proteasomal degradation. Together with MDM2, XBP1-u then translocates into the nucleus and forms a tertiary complex with tumor suppressor p53 protein, leading to p53 ubiquitination/proteasomal degradation, and subsequently, the promotion of tumorigenesis [11].

Another recently found XBP1-u function is its regulation of vascular smooth muscle cells (VSMCs) phenotypic transition and vascular homeostasis. Under normal physiological conditions, XBP1-u binds to forkhead box O4 (FoxO4) in the cytoplasm and suppresses its translocation into the nucleus, while during injury-induced ER stress, in which XBP1-u decreases due to the increase of its splicing, FoxO4 translocates into the nucleus. Accumulation of FoxO4 in the nucleus suppresses VSMCs’ differentiation, as it binds to and suppresses the transcriptional activity of myocardin, a transcriptional coactivator that regulates the transcription of various genes involved in VSMCs, such as *smooth muscle actin* (*SMA*), *calponin*, and *smooth muscle 22α* (*SM22α*) [12]. A very recent study further revealed that XBP1-u could not only maintain vascular homeostasis, but also inhibit vascular calcification. Under normal physiological conditions, XBP1-u binds to β-catenin and promotes its ubiquitination/proteasomal degradation, thereby suppressing its translocation into the nucleus. This in turn inhibits β-catenin/T cell factor (TCF)-mediated osteogenic markers *runt-related transcription factor 2* (*Runx2*) and *msh homeobox2* (*Msx2*) transcription, and subsequently, suppresses vascular calcification [13].

**Figure 2:**
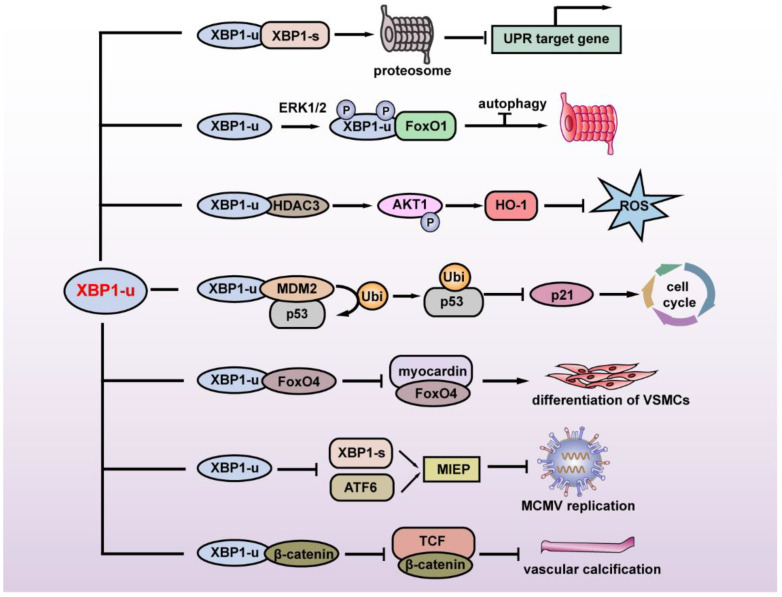
Summary of the physiological and pathological functions of XBP1-u. XBP1-u mediates post-translational modifications and plays a role in maintaining ER homeostasis, tumor development, oxidative stress, cell differentiation, vascular calcification, and viral infection. MIEP: major immediate-early promoter; MCMV: mouse cytomegalovirus; VSMCs: vascular smooth muscle cells.

This evidence shows that XBP1-u is not merely a precursor of XBP1-s, and instead has its own biological and pathological roles by regulating its target genes at the post-translational stage (Figure 2). The lack of the transcriptional activator domain and NLS at its C terminus define its cellular localization and specific regulatory pathway as distinct from the transcriptional regulation of XBP1-s.

## 3. Roles of XBP1 in Regulating Physiological Functions

### 3.1. The Role of XBP1 in ER Stress

**Figure 3:**
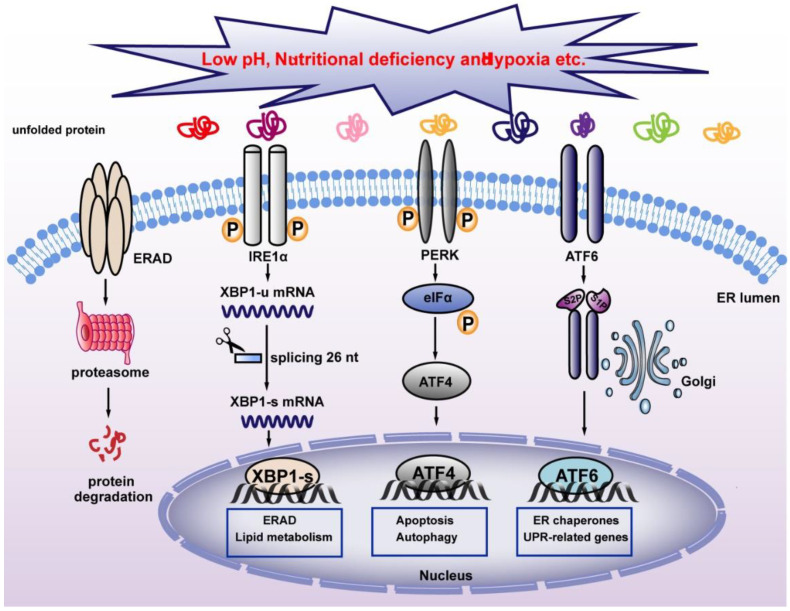
Classical pathway for maintaining ER homeostasis. Unfolded proteins activate UPR sensors IRE1α, PERK, and ATF6, which regulate UPR-associated target genes and maintain ER homeostasis. XBP1-s regulates ERAD and alleviates ER stress. IRE1α: inositol-requiring protein 1α; PERK: PERK-like ER kinase; ATF6: activating transcription factor 6; ERAD: ER-associated protein degradation.

Severe cellular environments, including nutrient deficiency, low pH, accumulation of unfolded or misfolded protein, and hypoxia could induce ER stress and thereby disrupt ER homeostasis. This will trigger UPR, a stress response that restores ER homeostasis to avoid apoptosis induced by strong ER stress (Figure 3). In mammalian cells, three ER transmembrane proteins, IRE1α, ATF6, and PERK operate as sensors of ER stress [43]. Both IRE1α and PERK belong to type I ER-resident transmembrane protein, which is the most evolutionarily conserved protein in the UPR pathways [44,45], while ATF6 is a type II ER-resident transmembrane protein with a cytoplasmic N-terminal b-ZIP domain [46]. Under normal conditions, BiP binds to these three sensors and inhibits their activation [16]. When ER stress occurs, BiP binds to misfolded proteins with higher affinity, and releases its binding with ER stress sensors, thereby activating them. PERK is the first sensor released upon exposure to ER stress. Through its serine/threonine kinase domain, activated PERK then phosphorylates eukaryotic translation initiation factor-2α (eIF2α) at Ser51. Phosphorylated eIF2α then induces systemic translation attenuation, including that of ATF4, which is crucial for ER homeostasis, thereby reducing the number of proteins entering the ER [47,48]. Meanwhile, activation of ATF6 leads to its translocation from the ER to the Golgi apparatus, where it is cleaved by the proteasome of site-1 protease (S1P) and site-2 protease (S2P), releasing its N-terminal fraction (ATF6 p50), which functions as a transcription factor regulating heat shock protein family A member 5(Hspa5), heat shock protein 90 β family member 1(Hsp90b1), and calreticulin, which are involved in protein folding and regulation of ER homeostasis [46]. IRE1α is the most conventional ER stress sensor. When ER stress occurs, IRE1α oligomerizes in the plane of the membrane and undergoes autophosphorylation at its RNase domain. This leads to its conformational changes and activation [49]. Activated IRE1α in turn mediates unconventional splicing of XBP1-u, resulting in the increase of XBP1-s. Unlike conventional splicing, which occurs in the nucleus and depends on the catalytic function of spliceosomes, XBP1-u splicing occurs in the cytoplasm and does not depend on spliceosomes, allowing its quick production in a time- and energy-saving manner [50]. XBP1-s then translocates into the nucleus and activates the transcription of various target genes involved in different stages of UPR, including *protein disulfide isomerase family A member 6* (*PDIA6*) and *SEC61 translocon subunit alpha 1* (*SEC61A1*), thereby alleviating ER stress. Furthermore, XBP1-s level regulates the transition between UPR-mediated survival and apoptosis. Under strong ER stress, increased XBP1-s upregulates the expression of *KLF9* by binding to its promoter. *KLF9* in turn transcriptionally activates the transmembrane protein 38B (TMEM38B) and inositol 1,4,5-trisphosphate receptor type 1 (ITPR1), resulting in the promotion of calcium release and cell death [23]. While severe ER stress leads to cell death, as will be explained in Section 4.4.1, a non-fatal ER stress response restores ER homeostasis, thereby facilitating cell adaptation to and survival of stress.

ER stress is negatively regulated by related factors and degradation mechanisms to maintain homeostasis. During ER stress, XBP1-s induces the expression of *MIST1*, which in turn induces negative feedback by directly binding to *XBP1-s* promoter and inhibiting its transcription. The decrease in XBP1-s relieves cellular pressure [24]. In addition, XBP1-s migrates to the nucleus and regulates the transcription of ER-associated protein degradation (ERAD) components to decrease ER stress [51].

Together, XBP1-s, as a splicing product of the UPR branch, plays different roles at different degrees of ER stress. Under strong ER stress, XBP1-s promotes cell death by changing calcium levels, while under non-fatal ER stress, XBP1-s reduces ER stress and promotes cell survival by transcriptionally regulating UPR target genes to increase ER protein folding capacity and promote the degradation of misfolded protein. In addition, XBP1-s regulates ER function and maintains intracellular homeostasis through positive and negative feedback regulation.

### 3.2. XBP1 Regulation of Metabolism

#### 3.2.1. XBP1 Induces Adipogenesis and Cholesterol Synthesis

Lipids are crucial molecules involved in maintaining biological pathways and cellular functions. They provide not only energy, but also sources of building blocks for cellular components, such as phospholipids that form the cell membrane and signaling molecules. Furthermore, lipids also affect the dynamics of cell division, senescence, and inflammation [52,53,54]. Lipid homeostasis is maintained through a balance of de novo lipid synthesis (fatty acid synthesis, FAS) and fatty acid oxidation (FAO), which leads to lipid degradation [55]. Deletion of XBP1 in mouse embryonic fibroblasts and adipocytes results in a serious defect in adipogenesis, indicating the crucial role of XBP1 in lipid metabolism. Early adipogenic factor CCAAT/enhancer-binding protein β (C/EBPβ) binds to the CCAG sequence at the proximal promoter of *XBP1-s*, and induces the expression of XBP1-s. Subsequently, XBP1-s binds to the promoter of the lipid-forming transcription factor *C/EBPα* and activates its gene expression to promote adipogenesis [29]. Furthermore, XBP1-s promotes adipogenesis by upregulating the expression of PPARγ2 [25]. During adipogenesis, the expression level of Wnt family member 10B (Wnt10b) decreases rapidly and thus acts as a molecular switch regulating fat formation. XBP1-s induces adipogenesis by inhibiting *Wnt10b* transcription and downregulating the anti-adipogenesis β-catenin pathway [56].

Liver is the main organ where lipid synthesis takes place. XBP1 deficiency significantly downregulates genes related to hepatic lipogenesis such as *diacylglycerol acetyltransferase 2* (*Dgat2*) and *acetyl CoA carboxylase 2* (*ACC2*), resulting in impaired de novo lipid synthesis [38]. Furthermore, the IRE1α/XBP1 pathway also plays an important role in insulin-mediated hepatic lipogenesis. Insulin disrupts the heterodimer formed by the P85α and P85β subunits of phosphoinositide 3-kinase (PI3K), a key component of insulin signal transduction. This in turn enhances the binding of P85α and P85β to XBP1-s, promotes XBP1-s nucleus translocation, and ameliorates ER stress [57]. Blocking the interaction between PI3K regulatory subunit and XBP1-s leads to lipid synthesis and lipid accumulation, and thus is the main cause of insulin resistance in obesity. Furthermore, XBP1-s also enhances insulin-mediated liver adipogenesis by activating the transcription of FAS-related genes, as well as that of the *sterol regulatory element binding protein* (*SREBP-1*) [26].

Hepatic very low-density lipoprotein (VLDL) is crucial for regulating intrahepatic and plasma lipid homeostasis. Elevated hepatic VLDL is a common cause of dyslipidemia. ER is the main site for lipid synthesis and VLDL assembly. The IRE1α/XBP1-s axis regulates cholesterol biosynthesis synthesis and secretion, especially of VLDL. Wang et al. revealed that IRE1α deficiency reduced PDI expression and triglyceride transfer protein (MTP) activity, leading to a VLDL assembly defect, while XBP1-s can induce PDI expression, thereby resolving VLDL assembly defects caused by IRE1α deletion [58].

Besides adipogenesis and cholesterol biosynthesis, XBP1-s also plays a key role in the formation of ER phospholipid membranes. XBP1-s promotes ER expansion by inducing the synthesis of phosphatidylcholine, the main phospholipid of ER, while knocking out *XBP1* reduces the fluidity of the ER membranes [59]. Together, XBP1-s is involved in various aspects of lipid metabolism, including adipogenesis, cholesterol assembly, and ER phospholipid synthesis.

#### 3.2.2. XBP1 Maintains Glucose Homeostasis by Regulating Insulin Metabolism

Insulin can promote the synthesis and metabolism of glycogen, lipids, and proteins. It is the key hormone for maintaining the balance of blood glucose homeostasis and energy storage. XBP1-s regulates insulin metabolism by regulating the functions of hepatocytes, pancreatic cells, and adipocytes.

Loss of insulin signaling in liver cells can lead to severe insulin resistance [60]. Zhou et al. demonstrated that XBP1-s was upregulated in the liver of both insulin-deficient and insulin-resistant mice, resulting in the degradation of FoxO1 through 26S proteasome. This in turn lead to an improved blood glucose level in model mice, as FoxO1 induces the increase of blood glucose by directly binding to and activating the promoters of gluconeogenic genes *phosphoenolpyruvate carboxykinase* (*PCK1*) and *glucose-6 phosphatase* (*G6PC*) [61,62,63]. XBP1-s also maintains blood glucose homeostasis by interacting with other insulin signaling-related mediators, such as p38 mitogen-activated protein kinase (p38 MAPK) and PI3K. On its Thr48 and Ser61, p38 MAPK phosphorylates XBP1-s. Together with the interaction of PI3K regulatory subunits with XBP1-s as mentioned above, these promote XBP1-s nuclear translocation and ameliorate ER stress [57,64]. Furthermore, Park et al. revealed that insulin enhances the formation of bromodomain-containing protein 7 (BRD7)/P85α/XBP1-s and BRD7/P85β/XBP1-s complexes, thereby promoting XBP1-s nuclear translocation and ultimately, reducing blood glucose levels in the obese and diabetic mouse models [65].

In pancreatic cells, XBP1-s regulates the secretion of insulin and glucagon. In pancreatic β cells, glucose stimulates XBP1-u splicing to expand the secretory capacity of the β cell for increased proinsulin synthesis. Moreover, XBP1-s binds directly to the promoter regions of PDI family genes, *PDI*, *PDIR*, *P5*, *ERp44*, and *ERp46*, thus increasing insulin and proinsulin production as well as proinsulin oxidative folding [30]. In pancreatic α cells, XBP1 deficiency causes glucose intolerance and insulin resistance due to dysregulated glucagon secretion. Knocking down *XBP1* increases IRE1α and Jun NH2-terminal kinase (JNK) phosphorylation, resulting in impaired insulin signaling, and subsequently, the inability of insulin to suppress glucagon secretion [66].

XBP1-s also regulates adipocyte insulin sensitivity. Cho et al. revealed that PPARγ2 improves systemic insulin sensitivity by regulating adipogenic and metabolic functions of mature adipocytes. XBP1-s increases PPARγ2 activity in adipocytes and enhances insulin-stimulated glucose uptake by promoting the expression of fibroblast growth factor 21 (FGF21), a PPARγ2-activating protein. Moreover, XBP1-s inhibits the secretion of pro-inflammatory adipokines by increasing insulin signal transduction and adiponectin secretion, and by eliminating palmitate-induced adipocyte insulin resistance [67,68].

Hence, XBP1-s is crucial for maintaining glucose homeostasis through suppressing glucogenesis, increasing insulin secretion and sensitivity, and promoting insulin-mediated glucose uptake.

### 3.3. The Roles of XBP1 in Developmental Biology

#### 3.3.1. XBP1 Regulates Follicular Development

In terms of germ cell development, XBP1 is closely related to follicular maturation. UPR is involved in follicular maturation, ovulation, and luteinization, as genes such as *XBP1-s*, *Hspa5*, *IRE1α*, and *PERK* are activated during follicular maturation. Furthermore, a previous study showed that XBP1-s level is significantly higher in cumulus cells enclosing oocytes receiving fertilization compared to those in enclosing oocytes without fertilization capacity [69]. XBP1-s is also crucial in regulating the estrus cycle and fertility by regulating the function of granulosa cells (GCs), which are key cells that promote follicular development by secreting estradiol. Knocking down *XBP1-s* not only promotes GC apoptosis, but also downregulates *hyaluronan synthase 2* (*Has2*) and *prostaglandin-endoperoxide synthase 2* (*Ptgs2*), key genes for follicular development. Furthermore, it reduces the expression of estrogen synthase cytochrome P450 family 19 subfamily A member 1 (CYP19A1), leading to the decrease of estradiol concentration, and subsequently, to follicle retardation [70,71].

#### 3.3.2. XBP1 Regulates the Development and Differentiation of Immune Cells

The immune system coordinates with other systems of the body to jointly maintain the stability of the internal environment and physiological balance. The immune system consists of multiple cells that orchestrate the whole cellular defense against pathogen invasion. XBP1-s plays important roles in immune system regulation by regulating the development and differentiation of various immune cells. We will discuss this issue in the following sections.

- Lymphocyte

Lymphocytes are the core of the immune response. According to their origins, morphological structures, surface markers, and immune functions, lymphocytes can be divided into T cells, B cells, and NK cells. Indeed, current studies clearly demonstrate that XBP1-s is involved with all three types of lymphocytes.

T cells are crucial for resistance against diseases, infections, and tumors. XBP1-s is involved in the regulation of both helper T cells (Th), which bind directly to the antigen via major histocompatibility complex MHC class I (MHCI) and damage the target cells, as well as cytotoxic T cells, which secrete cytokines to eliminate target cells [72,73]. Using genome-wide analysis, Pramanik et al. revealed that XBP1-s promotes Th2 cell differentiation by accelerating cell proliferation and enhancing Th2 cytokine production. The IRE1α/XBP1 pathway is activated during the activation of T cells and controls the expression of interleukin-13 (IL-13) and interleukin-5 (IL-5), two landmark cytokines of Th2 cells. Furthermore, it regulates Th2 cell proliferation by accelerating the S and G2/M phases, thereby promoting cell cycle progression [74].

XBP1-s is also involved in the differentiation of Th17 cells. Brucklacher et al. revealed that XBP1-s deficiency suppresses naive T cell differentiation towards Th17 cells, as well as the expression of interleukin-17 (IL-17) and retinoic acid receptor (RAR)-related orphan receptor γt (RORγt), two Th17 expression products which are associated with inflammatory and autoimmune diseases. Furthermore, cellular stress such as that induced by hypoxia, glucose deficiency, and osmotic stress continuously increases calcium levels and XBP1-s activities, resulting in the promotion of polarization and differentiation of naive T cells towards the Th17 cell subset [75].

Meanwhile, in response to acute infection, XBP1-s also promotes the differentiation of CD8+ T cells. During acute infection, pathogens such as bacteria or viruses activate the IRE1α/XBP1 pathway and promote XBP1-u splicing in CD8+ T cells. Increased XBP1-s upregulates killer cell lectin-like receptor G1 (KLRF1), which in turn promotes CD8+ T cell differentiation. Increased effector CD8+ T cells in turn quickly and efficiently resolve acute infections [76]. 

B cells activate and become plasma cells (PCs). PCs, which synthesize antibodies (immunoglobulins; Igs) are terminal cells that no longer have the ability to differentiate and proliferate. Iwakoshi et al. revealed that XBP1-s is involved in the production of interleukin-6 (IL-6), a cytokine essential for PC differentiation [77]. Furthermore, XBP1-s is crucial for antibody production and secretion. Under normal conditions, XBP1-s expression is increased during the differentiation of B cells into PCs. This in turn promotes Ig synthesis by inducing ER expansion. In XBP1-deficient lymphoid chimeric mice, while the proliferation and ability of B cells to form germinal centers are not affected, Ig synthesis is severely impaired. XBP1-s can also upregulate genes involved in Ig secretory pathways, including *signal recognition particle 54* (*SRP54*), *translocation-associated membrane protein 1* (*TRAM1*), and *multiple coagulation factor deficiency 2* (*MCFD2*) [70]. Altogether, XBP1-s is critical for the development of PCs, as well as for their ability to produce and secrete antibodies.

Natural killer (NK) cells are important media for host immunity against malignant tumors and viral infection, as they directly kill target cells and do not depend on antibodies. Dong et al. revealed that the IRE1α/XBP1-s pathway is upregulated in mouse cytomegalovirus (MCMV)-activated NK cells. Increased XBP1-s then promotes the expression level of c-Myc by binding to and activating *c-Myc* promoter. This results in the upregulation of c-Myc target gene, including *cyclin dependent kinase inhibitor 1A* (*CDKN1A*) and *heat shock protein family D member 1*(*Hspd1*), which are required for NK cell proliferation [78].

b. Monocytes, Macrophages and Dendritic cells (DCs)

Macrophages, monocytes, and DCs originate from hematopoietic stem cell-derived progenitors with myeloid-restricted differentiation potential, and XBP1-s is indeed involved in the maturation process of these lineages [79,80]. XBP1 splicing can be activated by pathogen sensors such as toll-like receptor 2 (TLR2) and toll-like receptor 4 (TLR4). Activated XBP1-s is in turn recruited to the promoters of inflammatory cytokines involved in innate immunity, such as *IL-6* and *tumor necrosis factor* (*TNF*) and enhances the innate response of macrophages by regulating their activities [81]. XBP1-s directly binds and enhances the transcription of *G-protein-coupled receptor 43* (*GPR43*), whose high expression can accelerate monocyte differentiation, indicating the possibility of XBP1-s enrollment in monocyte differentiation [82]. XBP1-s is also involved in maintaining the survival and development of DCs, and indeed, XBP1-s deficiency causes a decrease in the number of DCs and a deficiency in the highly elaborate rough ER with multiple layers in vivo, leading to a decrease in immune response [39]. Furthermore, XBP1-s also increases IFN-β expression in DCs, thereby enhancing their anti-viral effect [83].

c. Eosinophils

XBP1-s is an important factor in the regulation of the terminal differentiation of eosinophils. Bettigole et al. revealed that XBP1-s is selectively activated during the differentiation of eosinophils and is crucial for maintaining cell survival. Absence of XBP1-s leads to the disruption of ER homeostasis. This in turn causes a defect in key protein folding in newborn eosinophils and the weakening of secretory granule formation. Furthermore, these defects inhibit the expression of *GATA1*, a key factor in eosinophil development, resulting in differentiation arrest [40].

Hence, XBP1-s is crucial for the development of germ cells and the immune system. Furthermore, while its role in organ development is still not clear, XBP1-s deficiency causes hypoplastic fetal liver, resulting in a decrease in hematopoiesis and inducing anemia, and subsequently, embryonic lethality, indicating that XBP1-s might also play a crucial role in the development of other organs [84].

## 4. The Role of XBP1 in Diseases

### 4.1. XBP1 Regulates Inflammatory Disease

While XBP1-s and UPR are involved in the development of the immune system, aberrant activation of UPR is also involved in the pathogenesis of many inflammatory diseases. For instance, XBP1-s is positively correlated with the inflammation degree of chronic rhinosinusitis with nasal polyps (CRSwNPs), as it directly binds to the promoters of inflammatory factors *CCL5*, *IL1β*, and *IL-8*, promotes their transcriptions, and subsequently, aggravates the inflammatory response of CRSwNPs [7]. Furthermore, Dasgupta et al. demonstrated that activation of XBP1-s enhances the transcription of serine palmitoyltransferase, a key enzyme in ceramide synthesis, in nonalcoholic steatohepatitis (NASH) model mice, leading to the release of extracellular vesicles (EVs) and the recruitment of macrophages, which ultimately cause inflammation and liver injury [85].

Interestingly, XBP1-s can also reduce an inflammatory response. Inactivation of Sec63 homolog, protein translocation regulator (Sec63) in all distal nephron segments results in polycystin-1–mediated polycystic kidney disease (PKD). Ishikawa et al. developed a genetic mice model of tubulointerstitial kidney disease on the basis of inactivation of Sec63 and XBP1-s. The model presented symptoms of interstitial inflammation and progressive renal dysfunction. Meanwhile, ectopic expression of XBP1-s can reduce renal interstitial inflammation caused by the loss of Sec63 [86]. Moreover, XBP1-s deficiency induces spontaneous enteritis, resulting in impaired Paneth cell functions, including reduced production of anti-microbial peptides, as well as a reduced response to pathogens [87]. On the other hand, Yang et al. demonstrated that the absence of XBP1-s in Müller glial cells (MG) accelerates the occurrence of diabetic complications, as lack of XBP1-s increases retinal inflammation and vascular permeability in diabetic mice [88].

Altogether, XBP1-s plays a dual role in inflammatory diseases, as it can both inhibit and promote inflammation. The reason underlying these opposite effects remains unelucidated. However, as mentioned above, in responding to ER stress, the degree of ER stress determines the functions of XBP1-s in regulating cell survival; thus, it is necessary to investigate whether the degree of the immunostimulants, or whether they are acute or chronic stimulants, is involved in regulating these opposite roles. While XBP1-s might be a potential target for treating inflammation, more attention should be paid to avoid its opposite effect.

### 4.2. Neurodegenerative Disease

Neurodegenerative diseases are caused by the loss of neurons and their myelin sheath and include diseases that severely threaten both survival and quality of life, such as Alzheimer’s disease (AD), Parkinson’s disease (PD), Huntington’s disease (HD), amyotrophic lateral sclerosis (ALS), and different types of spinocerebellar ataxia (SCA).

AD is characterized by progressive and irreversible cognitive decline due to the abnormal formation and morphology of dendritic spines as well as a decrease in synaptic plasticity. The main causes include amyloid β (Aβ) accumulation, nerve fiber tangles, and damage to releasing neurotransmitters [89]. XBP1-s can ameliorate AD progression by driving the expression of ADAM10, thereby reducing Aβ plaque load and soluble Aβ [22,90]. Furthermore, as demonstrated by Martinez et al. using neural-specific XBP1 conditional knockout mice, XBP1 knockout significantly downregulated BDNF, which is crucial for neuronal growth and survival, while reintroduction of BDNF improved the synaptic plasticity and learning function of these mice. They also revealed that XBP1-s binds to the UPRE-II binding site in the proximal *BDNF* promoter region and directly regulates its transcription [31]. Furthermore, XBP1-s can also restore synaptic plasticity and memory function in AD models by enhancing the activity of *Kalirin-7* (*Kal7*), which is disrupted in AD, and promoting its expression level [37].

XBP1-s deficiency protects against HD and ALS by increasing autophagy. HD is caused by the glutamine amplification of Huntingtin (Htt) protein with more than 40 repeats. Mutant Htt accumulates in the form of an inclusion body, resulting in neuronal loss in the striatum. XBP1-s deficiency induces the expression of FoxO1, which in turn promotes autophagy and enhances mutant Htt clearance. These subsequently decrease the level of mutant Htt, and thus reduce the loss of neurons and improve motor ability [91]. XBP1-s regulation of autophagy is also associated with another neuronal disease, ALS, which is a progressive and fatal adult-onset motor neuron disease caused by mutations in gene-encoding *superoxide dismutase-1* (*SOD1*). More than 100 mutations that induce misfolding and abnormal aggregation, and subsequently motor neuron dysfunction in the gene encoding *SOD1*, are linked to ALS [92]. XBP1-s deficiency upregulates autophagy activity and thus enhances clearance of mutant SOD1 aggregates, thereby significantly reducing the accumulation of misfolded proteins, slowing the progression of ALS, and prolonging the life span of mutant *SOD1* transgenic mice [93].

Notably, as described above, a recent study revealed that XBP1-u is also involved in autophagy regulation in a mechanism different from that of XBP1-s. XBP1-u interacts with FoxO1 and recruits FoxO1 to the 20S proteasome for degradation, thereby inhibiting autophagy [9]. Thus, while further investigations are still needed, this finding indicates the possible role of XBP1-u on neurodegenerative diseases.

### 4.3. Virus Infection

When a virus infects a host cell, it constantly exerts pressure on the host cell. The newly synthesized glycoprotein of the progeny virus accumulates in the ER, depletes the folding ability of the ER, and leads to UPR activation. IRE1α/XBP1 are a double-edged sword in the process of virus replication, as they can not only promote virus replication, but can also exert an anti-viral function.

During influenza A virus (IAV) infection, ER stress is triggered and XBP1-s increases. XBP1-s downregulates the expression level of Sp1, a key controller of Cu-Zn superoxide dismutase SOD1, resulting in an increase of ROS. The virus titer increased with the increase of ROS; hence, XBP1-s finally exacerbated the viral infection [94]. The IRE1α/XBP1 pathway can also ensure the smooth progress of the replication of other viruses, including Zika virus, which can cause fetal congenital defects, and Marburg virus, which causes fatal hemorrhagic fever [8,95]. Furthermore, Johnston et al. demonstrated that Kaposi’s Sarcoma-associated Herpesvirus reduces XBP1-s production by inhibiting UPR sensor IRE1α, thereby promoting viral replication [96].

Interestingly, XBP1-s also plays anti-viral roles. Dengue virus infection activates the IRE1α/XBP1 pathway, thereby triggering ER stress-mediated apoptosis and alleviating the cytotoxicity of viral infection [97]. Sharma et al. also demonstrated that XBP1-s inhibits the replication of Japanese encephalitis virus by increasing the expression of *autophagy-related 3* (*ATG3*) and *Beclin 1*, which are crucial for inducing autophagy. An increase in autophagy in neuronal cells inhibits viral replication by decreasing viral titers and delaying virus-induced cell death [98].

Furthermore, recent studies reported that besides XBP1-s, XBP1-u can also exert anti-viral activity. In the early stage of MCMV infection, IRE1α-mediated XBP1 splicing is blocked due to the inhibition of IRE1α activity, resulting in an increase of XBP1-u. XBP1-u in turn blocks the activation of the viral major immediate-early promoter (MIEP), leading to the inhibition of viral replication [99].

In conclusion, XBP1 has a dual role in response to viral infection, that is, promoting viral replication and playing an antiviral role. The reason for this contradictory response remains unclear, and further research is needed to unravel the mechanism underlying this paradoxical effect.

### 4.4. Cancer

Both XBP1-u and XBP1-s are highly expressed and can drive the development of a variety of tumors, including colorectal cancer, hepatocellular carcinoma, multiple myeloma, ovarian cancer, breast cancer, melanoma, and bladder cancer, by regulating various aspects of tumorigenesis (Table 2) [34,100,101,102,103,104,105]. Tumor cell microenvironments, such as hypoxia and nutrient deprivation, induce ER stress, thereby activating the UPR stress signaling pathways including the IRE1α/XBP1-s axis. This activation in turn triggers enhanced transcription of target genes, and subsequently drives tumor growth and progression by affecting cell proliferation, tumor metastasis, and immune system evasion, which are known as hallmarks of cancer [106,107]. Furthermore, recent studies revealed that XBP1-u is also crucial for tumorigenesis. In this section, we will describe the relationship between XBP1 and hallmarks of cancer.

#### 4.4.1. XBP1 Promotes Tumorigenesis

XBP1 can regulate tumor cell proliferation by suppressing tumor suppressor genes as well as by activating oncogenes. The p53 family is a well-known tumor suppressor gene family consisting of tumor suppressor p53, TAp73, and p63 [116]. In more than 50% of tumor patients, p53 mutations can be found,, while its deregulation occurs frequently in patients with wild-type gene [117]. Mutations in and aberrant expression of the p53 family result in a defect in their functions in suppressing tumorigenesis and tumor progression and are correlated with poor prognosis. XBP1-u can destabilize p53 protein by forming a complex with MDM2 and promoting p53 ubiquitination/proteasomal degradation. This leads to the downregulation of the p53/p21 axis, and thereby accelerates cell cycle progression and tumor cell proliferation. [11]. Meanwhile, XBP1 regulation of TAp73, another member of the p53 family, occurs at the transcriptional stage. In this regulation, XBP1-s binds to the TAp73 promoter and suppresses its transcriptional activity, leading to the enhanced proliferation of tumor cells [100]. As p53 is crucial not only for preventing abnormal cell proliferation, but also for suppressing other aspects of tumorigenesis, including tumor angiogenesis, metabolic reprogramming, tumor metastasis, and prevention of apoptosis, these facts indicate that the XBP1-u/MDM2/p53 axis might also be involved in the regulation of these hallmarks of cancers. Furthermore, as mentioned previously, XBP1-u also exerts its anti-tumor function by promoting FoXO1 degradation, thereby inhibiting tumor cells autophagy [9].

Current studies have revealed that activation of oncogenes by XBP1 occurs through XBP1-s-mediated transcriptional regulation. In breast cancer cells, UPR stimulation and estrogen can enhance the level of XBP1-s, which in turn promotes tumor cell proliferation by activating the transcription of *NCOA3* as well as the Rab9/PIK/Akt pathway [35,36]. XBP1-s can also bind to the ACGT core sequence of the *IL-6* promoter and enhance its transcription, leading to the activation of the STAT3 signaling pathway and enhanced tumorigenesis of melanoma, hepatocarcinoma, and primary effusion lymphoma [34,105,108]. XBP1-s also increases the transcription of c-Myc as well as PRKAR2B, the regulatory subunit of the protein kinase A, and enhances prostate cancer cell proliferation [109].

Adaptation to tumor microenvironments such as hypoxia is crucial for increasing tumor cell survival and promoting tumor cell proliferation, and XBP1 is indeed involved in this regulation [118]. XBP1 splicing is activated upon exposure to hypoxia [119]. Increased XBP1-s promotes glycolysis by positively regulating the expression of hexokinase 2 (HK2) and enhances tumor cell viability under hypoxia [120]. Moreover, hypoxia-induced ER stress leads to the downregulation of low-density lipoprotein receptor-related protein 6 (LRP6), which reduces the stability of β-catenin. This results in XBP1-s augmentation of hypoxia-inducible factor 1α (HIF-1α) transcriptional activity and subsequently, promotion of tumor cell survival [121]. Besides, XBP1-s can also regulate the HIF-1α pathway by forming a transcription complex with HIF-1α and regulating the expression of HIF-1α targets, including *vascular endothelial growth factor A* (*VEGFA*), *pyruvate dehydrogenase kinase 1* (*PDK1*), and *glucose transporter 1* (*GLUT1*). Subsequently, it promotes the development of triple-negative breast cancer (TNBC) [102].

Regulation of redox homeostasis is also crucial for tumor cell survival. Due to their rapid proliferation, tumor cells produce a large number of ROS, whose excess amount is harmful for tumor cells. Liu et al. demonstrated that XBP1-s exerts a crucial role for tumor cell antioxidant defense by upregulating antioxidant factors including thioredoxin-1 (TRX1), SOD1, and catalase, which in turn suppress ROS and promote glioma cells survival [110]. Meanwhile, Song et al. showed that *XBP1* silencing activates the Mst1 pathway and promotes the phosphorylation of c-Jun N-terminal kinase (JNK). Activation of the Mst1/JNK pathway mediates excessive production of mitochondrial-reactive oxygen species, which are lethal for tumor cells [122].

Furthermore, XBP1-s can also promote tumor development by modulating immune cells. During the development of ovarian cancer, activation of the IRE1α/XBP1-s pathway by glucose deficiency-induced ER stress suppresses the expression of glutamine transporters’ solute carrier family 1 member 5 (SLC1A5), solute carrier family 38 member 1 (SLC38A1), and solute carrier family 38 member 2 (SLC38A2). These lead to the restriction of T cells’ glutamine uptake, thereby impairing T cells’ mitochondrial respiration and their anti-tumor function [104]. Furthermore, XBP1-s also drives the progression of ovarian cancer by disrupting DC function. Activation of XBP1-s by unsaturated aldehyde 4-hydroxy-trans-2-nonenal (4-HNE), a byproduct of lipid peroxidation, promotes the production of triglyceride and aberrant lipid accumulation in DCs, and subsequently, causes the failure of DCs to assist T cells in exerting their anti-tumor function [123].

In general, these studies show the prominent role of XBP1 in promoting tumor cell proliferation and reveal the regulatory mechanism of XBP1 at the transcriptional and post-translational levels. It is notable that although tumorigenic functions of XBP1-u, besides promoting tumor cell proliferation through suppression of tumor suppressors, have not been reported, it is highly probable that XBP1-u is also involved in the regulation of oncogenes and other hallmarks of cancer, as it can modulate post-translational regulation, including phosphorylation and ubiquitination, which are crucial for regulating the activity and stability of a large number of proteins.

#### 4.4.2. XBP1 and Cancer Metastasis

Tumor metastasis is responsible for poor prognosis and a 90% solid tumor mortality rate [124]. Epithelial–mesenchymal transition (EMT), characterized by the loss of epithelial characteristics and the acquisition of mesenchymal characteristics, is considered to be the first step in metastasis [41]. Previous studies reported that in breast cancer, lung cancer, oral squamous cell carcinoma, and hepatocellular carcinoma, XBP1-s promotes metastasis by directly upregulating the transcription of EMT-inducing factors, such as *Snail* and *zinc finger E-box* (*ZEB*), leading to poor prognosis and a low survival rate [42,111,112,125,126].

After obtaining the mesenchymal characteristics, tumor cells secrete proteases such as matrix metalloproteinase MMPs to degrade extracellular matrix (ECM) and promote tumor cell invasion. Luo et al. reported that XBP1-s promotes MMP-9 expression by upregulating insulin-like growth factor-binding protein-3 (IGFBP3). MMP-9 in turn enhances cell invasion and metastasis by digesting ECM and intercellular adhesion proteins [113,127]. Furthermore, XBP1-s can directly regulate ECM remodeling and promote thyroid cancer metastasis [128].

Finally, XBP1-s is negatively correlated with poor prognosis and low survival of pulmonary adenocarcinoma, colon carcinoma, and gallbladder cancer patients [114,115,129], further supporting the view that increased XBP1-s expression contributes to the acquisition of EMT phenotype and tumor metastasis. Interestingly, a recent report by Yang et al. showed that XBP1-s can inhibit EMT in papillary thyroid cancer [130]; however, whether this regulation occurs in specific cancers or applies more generally remains unknown. Furthermore, the molecular mechanism underlying this discrepancy also needs to be investigated.

#### 4.4.3. XBP1 Correlates with Tumor Drug Resistance

Drug resistance is one of the largest hurdles for anti-tumor therapeutics. XBP1-s is also involved in the increase of tumor cells’ drug resistance. For instance, 5-fluorouracil (5-FU), a major anti-tumor drug used for treating various tumors including colon carcinoma, esophageal squamous cell carcinoma, and breast cancer, induces the activation of the IRE1α/XBP1 pathway, leading to the upregulation of ATP binding cassette (ABC) transporters, which are crucial for drug resistance [131]. Furthermore, tamoxifen, another major anti-tumor drug for treating breast cancer, can also increase XBP1 mRNA expression in drug-resistant breast cancer cells. [132]. XBP1-s downregulation in these cancer cells can increase the inhibitory effect of estrogen receptor antagonists; hence, XBP1-s is a potential target for overcoming the tumor cells’ drug resistance problem. Indeed, small molecules targeting the XBP1-s pathway have been reported as potential anti-tumor therapeutic agents. STF-083010, which suppresses XBP1 splicing by blocking the activity of IRE1α, can increase the sensitivity of tumor cells to bortezomib and tamoxifen [133]. Moreover, Chen et al. revealed that suppressing the interaction between XBP1-s and the promoter region of β-catenin using CYD6-17, an oridonin analogue, can efficiently reduce the expression of β-catenin, and subsequently suppress the growth of the drug-resistant muscle-invasive bladder cancer cells [103].

## 5. Conclusions and Perspectives

**Figure 4:**
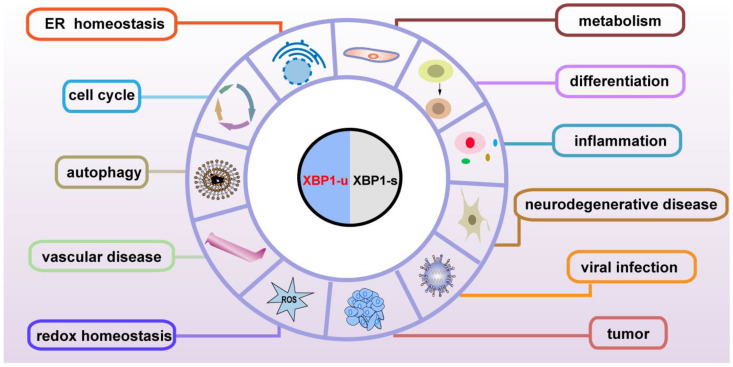
Pathophysiological functions of XBP1-u and XBP1-s.

Although the subcellular localization and the molecular mechanisms are different, both the spliced and unspliced XBP1 isoforms regulate various genes involved in physiological and pathological functions, including maintenance of ER homeostasis, metabolism, development, the immune system, neurodegeneration, cardiovascular protection, and tumor progression, through transcriptional and post-transcriptional regulation (Figure 4). XBP1-s binds to its specific binding sites on the promoter of the target genes, and thus regulates them at the transcriptional level (Table 3); meanwhile, XBP1-u regulates its targets at the post-translational level (Table 4). As XBP1-u can regulate target factors’ ubiquitination and phosphorylation, which are major post-translational modifications of proteins, it is highly probable that the functions of XBP1-u are still underestimated and hence need to be further elucidated. Indeed, XBP1-u is converted to XBP1-s under ER stress. Whether there are other mechanisms driving the conversion of XBP1-u to XBP1-s remains totally unclear. The detailed different roles of XBP1-u and XBP1-s in pathophysiology still need to be further studied.

Due to their broad physiological and pathological functions, both XBP1-s and XBP1-u have attracted attention as targets for treating many diseases, including neurodegenerative diseases, inflammatory diseases, viral infection and tumors. While mounting evidence shows that XBP1 is not only crucial for ER stress, further studies are necessary to unravel several unsolved questions regarding the functions of XBP1, including the functions of XBP1-u, as well as the degree of XBP1-u’s contribution to regulating the same phenotype in cells having both XBP1-u and XBP1-s, for instance, in tumor cells. Furthermore, targeting XBP1-s and XBP1-u have become novel, promising therapeutic strategies. However, the mechanism underlying the contradictive functions of XBP1 in regulating cell survival as well as in responding to viral infection remains unelucidated, and thus a thorough investigation regarding these opposite effects is absolutely necessary before these strategies can be applied clinically, either alone or in combination with other drugs.

Another hurdle for therapeutic strategies targeting XBP1-u and XBP1-s is their high homology, especially on nucleotide sequences. As they only differ at the +541 to +566 nucleotides of XBP1-u, which are excised during splicing to form XBP1-s, it is hard to target them specifically, for example, by using RNA interference. Hence, targeting the C termini of their proteins, which are totally different due to a codon shift during splicing, or delivering inhibitors specifically to the cytosol where XBP1-u localizes, or to the nuclei where XBP1-s localizes, might become potential choices to specifically interfere with the expression of XBP1 isoforms.

Overall, while further investigations on their molecular mechanisms need to be carried out, the broad scope of the functions of XBP1 isoforms have revealed their importance in maintaining various physiological functions, thus highlighting their potential as prognostic markers and therapeutic targets.

## Figures and Tables

**Table 1:** Direct targets of XBP1-s.

Gene	Regulation	Binding Motif	Physiological/Pathological Functions	Ref
*ADAM10*	Activation	UPRE	Alzheimer’s disease	[22]
*KLF9*	Activation	UPRE	ER homeostasis	[23]
*MIST1*	Repression	UPRE	Cellular pressure	[24]
*BDNF*	Activation	UPRE-II	Learning and memory function	[31]
*GRP94*	Activation	ERSE	Cell survival	[27]
*BiP*	Activation	ERSE	Protein folding	[4]
*calreticulin*	Activation	ERSE	Protein folding	[4]
*PPARγ2*	Activation	ERSE	Adipogenesis	[25]
*SREBP-1*	Activation	ERSE	Hepatic adipogenesis	[26]
*Herpud1*	Activation	ERSE- II	Protein degradation	[3]
*ARMET*	Activation	ERSE- II	Protein quality control	[28]
*P5*	Activation	ACGT	Insulin production	[30]
*PDI*	Activation	ACGT	Insulin production	[30]
*PDIR*	Activation	ACGT	Insulin production	[30]
*ERp44*	Activation	ACGT	Insulin production	[30]
*ERp46*	Activation	ACGT	Insulin production	[30]
*C/EBPα*	Activation	ACGT	Adipogenesis	[29]
*IL-6*	Activation	ACGT	Tumor cell proliferation	[34]
*NCOA3*	Activation	ACGT	Tumor cell proliferation	[35]
*PRKAR2B*	Activation	ACGTCA	Tumor cell proliferation	[33]
*Rab9*	Activation	ACGTCA	Tumor cell proliferation	[36]
*ATF4*	Activation	AGGGAGACGGCAGC	Tumor cell proliferation	[32]
*Kal7*	Activation	n/a	Synaptic plasticity	[37]
*IL-8*	Activation	n/a	Inflammatory response	[7]
*CCL5*	Activation	n/a	Inflammatory response	[7]
*IL-1β*	Activation	n/a	Inflammatory response	[7]
*IFN-γ*	Activation	n/a	Inflammatory response	[7]
*Dgat2*	Activation	n/a	Hepatic adipogenesis	[38]
*Acc2*	Activation	n/a	Hepatic adipogenesis	[38]
*c-Myc*	Activation	n/a	NK cell proliferation	[39]
*GATA1*	Activation	n/a	Eosinophil differentiation	[40]
*Snail*	Activation	n/a	Tumor metastasis	[41]
*ZEB*	Activation	n/a	Tumor metastasis	[42]

Abbreviations: ER: endoplasmic reticulum; ERSE: endoplasmic reticulum stress response element; ERSE-II: endoplasmic reticulum stress response element; NK: natural killer; n/a: not available; UPRE: unfolded protein response element; UPRE-II: unfolded protein response element.

**Table 2:** Expression and role of XBP1 in various cancers.

Cancer Types	Expression	Prognosis	Function	Ref
Colorectal cancer	Upregulated	n/a	Promote cell proliferation	[11]
Multiple myeloma	Upregulated	Poor	Promote cell proliferation	[101]
Melanoma	Upregulated	n/a	Promote tumor development	[34]
Bladder cancer	Upregulated	Poor	Promote cell proliferation	[103]
Ovarian cancer	Upregulated	Poor	Damage T cell function	[104]
Hepatocellular carcinoma	Upregulated	Poor	Promote cell proliferation	[105]
Breast cancer	Upregulated	Poor	Promote cell proliferation	[36]
Primary effusion lymphoma	Upregulated	n/a	Promote cell survival	[108]
Prostate cancer	Upregulated	Poor	Promote tumor development	[109]
Glioma	Upregulated	n/a	Promote cell survival	[110]
Non-small cell lung cancer	Upregulated	Poor	Promote metastasis	[111]
Oral squamous cell carcinoma	Upregulated	Poor	Promote metastasis	[112]
Pulmonary adenocarcinoma	Upregulated	Poor	Promote tumor development	[113]
Gallbladder cancer	Upregulated	Poor	Promote metastasis	[114]
Papillary thyroid cancer	Downregulated	Good	Inhibit EMT	[115]

Abbreviations: EMT: epithelial–mesenchymal transition; n/a: not available.

**Table 3:** Biological functions of XBP1-s.

Functions	Related Genes	Effect	Ref
ER homeostasis	*KLF9*	Maintain ER homeostasis	[23]
	*MIST1*	Relieve cellular pressure	[24]
Lipid metabolism	*C/EBPα*	Promote adipogenesis	[29]
	*PPARγ2*	Promote adipogenesis	[25]
	*SREBP-1*	Promote adipogenesis	[26]
	*Wnt10b*	Promote adipogenesis	[56]
Insulin metabolism	*PDI*	Increase insulin synthesis	[30]
	*FoxO1*	Maintain glucose homeostasis	[61]
	*PPARγ2*	Enhance glucose uptake	[67]
Development	*Has2*	Promote follicle maturation	[70]
	*Ptgs2*	Promote follicle maturation	[70]
	*IL-6*	Promote plasma cell differentiation	[73]
	*IL-5*	Promote Th2 cell differentiation	[76]
	*IL-13*	Promote Th2 cell differentiation	[76]
	*IL-17*	Promote Th17 cell differentiation	[77]
	*RORγt*	Promote Th17 cell differentiation	[77]
	*KLRF1*	Promote CD8+ T cell differentiation	[134]
	*c-Myc*	Promote NK cell proliferation	[81]
	*GATA1*	Promote eosinophil differentiation	[40]
Inflammatory response	*IL-8*	Enhance inflammation	[7]
	*CCL5*	Enhance inflammation	[7]
Memory function	*ADAM10*	Ameliorate the progression of AD	[22]
	*BDNF*	Restore synaptic plasticity	[31]
	*Kal7*	Restore synaptic plasticity	[37]
Motor function	*FoxO1*	Improve motor ability	[91]
	*SOD1*	Slow the progression of ALS	[93]
Antiviral	*ATG3*	Inhibit virus replication	[98]
	*Beclin1*	Inhibit virus replication	[98]
Oncogene	*TAp73*	Promote cell proliferation	[100]
	*HIF-1α*	Promote cell proliferation	[102]
	*IL-6*	Promote cell proliferation	[34]
	*NCOA3*	Promote cell proliferation	[35]
	*Rab9*	Promote cell proliferation	[36]
	*c-Myc*	Promote cell proliferation	[109]
	*HK2*	Promote cell proliferation	[120]
	*TRX1*	Promote cell proliferation	[110]
Immune escape	*SLC1A5*	Impair immune cell function	[104]
	*SLC38A2*	Impair immune cell function	[104]
	*SLC38A5*	Impair immune cell function	[104]
Tumor metastasis	*Snail*	Promote metastasis	[41]
	*ZEB*	Promote metastasis	[42]
	*MMP9*	Promote metastasis	[127]

Abbreviations: AD: Alzheimer’s disease; ALS: amyotrophic lateral sclerosis; ER: endoplasmic reticulum; NK: natural killer.

**Table 4:** Biological functions of XBP1-u.

Related Functions	Target	Pathway	Modification	Ref
Autophagy	FoxO1	XBP1-u/FoxO1	Ubiquitination	[9]
Redox homeostasis	Akt1	HDAC3/Akt1/Nrf2	Phosphorylation	[10]
Cell cycle regulation	P53	MDM2/p53/p21	Ubiquitination	[11]
Vascular homeostasis	FoxO4	FoxO4/myocardin	n/a	[12]
Vascular calcification	β-catenin	β-catenin/Runx2/Msx2	Ubiquitination	[13]
ER homeostasis	XBP1-s	IRE1α/XBP1	n/a	[14]
Anti-viral defense	MIEP	IRE1α/XBP1	n/a	[99]

Abbreviations: MIEP: major immediate-early promoter; n/a: not available.

## Data Availability

Not applicable.

## Notes

Conceptualization: V.K. and S.W.; investigation: X.L., L.A. and M.W.; writing—original draft preparation: V.K., S.W. and X.L.; writing—review and editing: V.K., S.W., X.L., L.A. and M.W. All authors have read and agreed to the published version of the manuscript.

## Abbreviations

ABC: ATP binding cassette; ACC2: acetyl CoA carboxylase 2; AD: Alzheimer’s disease; ADAM10: disintegrin and metalloproteinase 10; Akt1: Akt serine/threonine kinase 1; ALS: amyotrophic lateral sclerosis; ATF4: activating transcription factor 4; ATF6: activating transcription factor 6; ATG3: autophagy related 3; ARMET: arginine rich, mutated in early stage of tumors; Aβ: amyloid β; BDNF: brain-derived neurotropic factor; BiP: binding immunoglobulin protein; BHLHA15: basic helix–loop–helix family member a15; BRD7: bromodomain-containing protein 7; bZIP: basic leucine zipper; CCL5: chemokine ligand 5; C/EBPα: CCAAT/enhancer-binding protein α; C/EBPβ: CCAAT/enhancer-binding protein β; CRSwNPs: chronic rhinosinusitis with nasal polyps; CYP19A1: estrogen synthase cytochrome P450 family 19 subfamily A member 1; D-Flow: disturbed flow; Dgat2: diacylglycerol acetyltransferase 2; ECM: extracellular matrix; eIF2α: eukaryotic translation initiation factor-2α; EMT: epithelial–mesenchymal transition; ER: endoplasmic reticulum; ERAD: ER-associated protein degradation; ERK1/2: protein kinase 1/2; ERp44: endoplasmic reticulum protein 44; ERp46: endoplasmic reticulum protein 46; ERSE: endoplasmic reticulum stress response element; ERSE-II: endoplasmic reticulum stress response element II; EVs: extracellular vesicles; FAO: fatty acid oxidation; FAS: fatty acid synthase; FGF21: fibroblast growth factor 21; FoxO1: forkhead box O1; FoxO4: forkhead box O4; 5-FU: 5-fluorouracil; gB: glycoprotein B; GCs: granulosa cells; GLUT1: glucose transporter 1; GPR43: G-protein-coupled receptor 43; GRP94: glucose-regulated protein 94; G6PC: glucose-6 phosphatase; Has2: hyaluronan synthase 2; HD: Huntington’s disease; HDAC3: histone deacetylase 3; Herpud1: homocysteine inducible ER protein with ubiquitin-like domain 1; HIF-1α: hypoxia-inducible factor 1α; HK2: hexokinase 2; Hspa5: heat shock protein family A member 5; Hspd1: heat shock protein family D member 1; Hsp90b1: heat shock protein 90 β family member 1; HO-1: heme oxygenase 1; Htt: Huntingtin; 4-HNE: 4-hydroxy-trans-2-nonenal; IAV: influenza A virus; IE1: immediate-early 1; IFN-β: interferon β; Ig: immunoglobulins; IGFBP3: insulin-like growth factor-binding protein-3; IL-6: interleukin-6; IL-8: interleukin-8; IL-17: interleukin-17; IRE1α: inositol-requiring protein 1α; ITPR1: inositol 1,4,5-trisphosphate receptor type 1; JNK: c-junn-terminal kinase; KLF9: Krüppel-like factor 9; KLRF1: killer cell lectin-like receptor G1; LRP6: lipoprotein receptor-related protein 6; MCFD2: multiple coagulation factor deficiency 2; MCMV: mouse cytomegalovirus; MDM2: mouse double-minute 2 homolog; MG: Müller glial cells; MHC: major histocompatibility complex; MIEP: major immediate-early promoter; MTP: triglyceride transfer protein; mTORC2: rapamycin complex 2; Msx2: msh homeobox 2; NASH: nonalcoholic steatohepatitis; NF-Y: nuclear factor Y; NK: natural killer; Nrf2: NF-E2-related factor 2; PCs: plasma cells; PCK1: phosphoenolpyruvate carboxykinase; PD: Parkinson’s disease; PDI: protein disulfide isomerase; PDIA3: protein disulfide isomerase family A member 3; PDIA6: protein disulfide isomerase family A member 6; PDIR: protein disulfide isomerase related protein; PDK1: pyruvate dehydrogenase kinase 1; PERK: PERK-like ER kinase; PI3K: phosphoinositide 3-kinase; PKD: polycystic kidney disease; PPARγ2: peroxisome proliferator-activated receptor γ2; Ptgs2: prostaglandin-endoperoxide synthase 2; PRKAR2B: protein kinase cAMP-dependent type II regulatory subunit β; P5: protein disulfide isomerase P5; p38 MAPK: p38 mitogen-activated protein kinase; ROS: reactive oxygen species; RORγt: retinoic acid receptor (RAR)-related orphan receptor γt; Runx2: runt-related transcription factor 2; SCA: spinocerebellar ataxia; SEC61A1: SEC61 translocon subunit alpha 1; Sec63: Sec63 homolog, protein translocation regulator; SLC1A5: solute carrier family 1 member 5; SLC38A1: solute carrier family 38 member 1; SLC38A2: solute carrier family 38 member 2; SMA: smooth muscle actin; SM22α: smooth muscle 22α; SOD1: superoxide dismutase-1; SREBP-1: sterol regulatory element binding transcription factor 1; SRP54: signal recognition particle 54; S1P:site-1 protease; S2P:site-2 protease; TCF: T cell factor; Th: helper T cells; TLR2: toll-like receptor 2; TLR4: toll-like receptor 4; TMEM38B: transmembrane protein 38B; TNBC: triple-negative breast cancer; TNF: tumor necrosis factor; TRAM1: translocation-associated membrane protein 1; TRX1: thioredoxin-1; UPR: unfolded protein response; UPRE: unfolded protein response element; VEGFA: vascular endothelial growth factor A; VLDL: very low-density lipoprotein; VSMCs: vascular smooth muscle cells; XBP1: X-box binding protein 1; XBP1-s: spliced XBP1; XBP1-u: unspliced XBP1; ZEB: zinc finger E-box.
